# Elasto-mammography: Theory, Algorithm, and Phantom Study

**DOI:** 10.1155/IJBI/2006/53050

**Published:** 2006-02-01

**Authors:** Z. G. Wang, Y. Liu, L. Z. Sun, G. Wang, L. L. Fajardo

**Affiliations:** ^1^ Department of Civil and Environmental Engineering, The University of Iowa, Iowa City, IA 52242, USA; ^2^ Department of Radiology, The University of Iowa, Iowa City, IA 52242, USA; ^3^ Department of Civil and Environmental Engineering, University of California, Irvine, CA 92697, USA

## Abstract

A new imaging modality framework, called elasto-mammography, is proposed to generate the elastograms of breast tissues based on conventional X-ray mammography. The displacement information is extracted from mammography projections before and after breast compression. Incorporating the displacement measurement, an elastography reconstruction algorithm is specifically developed to estimate the elastic moduli of heterogeneous breast tissues. Case studies with numerical breast phantoms are conducted to demonstrate the capability of the proposed elasto-mammography. Effects of noise with measurement, geometric mismatch, and elastic contrast ratio are evaluated in the numerical simulations. It is shown that the proposed methodology is stable and robust for characterization of the elastic moduli of breast tissues from the projective displacement measurement.


Hindawi Publishing Corporation
International Journal of Biomedical Imaging
Volume 2006, Article ID 53050, Pages 1–11
DOI 10.1155/IJBI/2006/53050

### Elasto-mammography: Theory, Algorithm, and Phantom Study

**Z. G. Wang,[1] Y. Liu,[2] L. Z. Sun,[3] G. Wang,[2] and L. L. Fajardo[2]**

[1] *Department of Civil and Environmental Engineering, The University of Iowa, Iowa City, IA 52242, USA*
[2] *Department of Radiology, The University of Iowa, Iowa City, IA 52242, USA*
[3] *Department of Civil and Environmental Engineering, University of California, Irvine, CA 92697, USA*

Received 4 September 2005; Accepted 7 November 2005

Recommended for Publication by Jie Tian

A new imaging modality framework, called elasto-mammography, is proposed to generate the elastograms of breast tissues based on conventional X-ray mammography. The displacement information is extracted from mammography projections before and after breast compression. Incorporating the displacement measurement, an elastography reconstruction algorithm is specifically developed to estimate the elastic moduli of heterogeneous breast tissues. Case studies with numerical breast phantoms are conducted to demonstrate the capability of the proposed elasto-mammography. Effects of noise with measurement, geometric mismatch, and elastic contrast ratio are evaluated in the numerical simulations. It is shown that the proposed methodology is stable and robust for characterization of the elastic moduli of breast tissues from the projective displacement measurement.

Copyright © 2006 Z. G. Wang et al. This is an open access article distributed under the Creative Commons Attribution License, which permits unrestricted use, distribution, and reproduction in any medium, provided the original work is properly cited.

### 1. INTRODUCTION

Breast cancer is one of the major threats to public health in the world. Approximately 10% of women will develop breast cancer during the course of their lives in USA and Europe. The specific causes of breast cancer are yet unknown. Therefore early detection of breast tumor is the key to successful treatment.

X-ray mammography is the primary method for early detection of breast cancers [1]. According to the reports of US Food and Drug Administration, mammography can find 85 to 90 percent of breast cancers in women over 50, and can detect a lump up to two years before it can be sensed by manual palpation. While effective for detecting breast abnormality, mammography is not quite specific for differentiating benign and malignant masses, especially when the breast tissue is radiodense. A significant number of suspicious masses identified by mammography for surgical breast biopsy are in fact not malignant [2]. False-positive mammograms induce anxiety, distress, and intrusive thoughts.

It has been well recognized that the tissue stiffness plays an important role in diagnosis of breast cancers, as tumors are stiffer than the surrounding breast tissues [3, 4], and malignant tumors are much stiffer than benign ones [5]. In other words, in vivo identification of the elastic moduli of normal and abnormal breast tissues, which describe the stiffness, should improve the accuracy of breast cancer diagnosis. There have been elastography studies based on either ultrasound or MRI breast imaging [6–12]. Ophir et al. [6, 7] and Souchon et al. [8] proposed an ultrasound elastography modality for quantitative imaging of the elastic modulus distributions in biological tissues. Muthupillai et al. [9] and Manduca et al. [10] developed an algorithm to reconstruct the shear modulus distribution using acoustic strain wave propagation measured with MRI technique. Plewes et al. [11] and Samani et al. [12] provided a finite-element iteration method to reconstruct the distribution of elastic moduli in a breast containing suspicious tumors, based on the MRI deformation measurement under compression loading.

The objective of this study is to develop a new imaging modality, called elasto-mammography, for quantification of the elastic moduli of normal and cancerous breast tissues. In contrast to the previous breast elastography developments, elasto-mammography does not require additional biomedical imaging measurements and extra expense; that is, it combines the conventional low-dose X-ray mammography directly with our previously proposed tomography-based

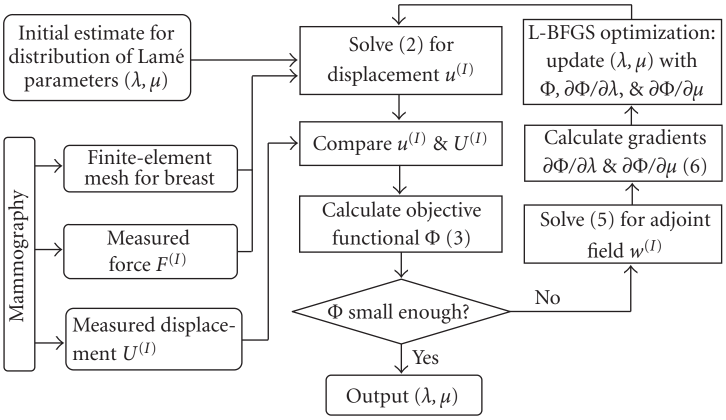

Figure 1: Overall flowchart for elasto-mammography reconstruction of Lamé parameters $\lambda$ and $\mu$ of breast tissues.

elastography framework [13]. Specifically, by adopting certain anatomically well-motivated assumptions, the geometry of tumors is estimated from the mammography projections, as well as from the displacements at key points. The elastography reconstruction is further conducted with our highly efficient algorithm for the elastic parameters of tissues.

This work is organized as follows. In Section 2, we recall our optimization-based algorithm for elastography reconstructions. We further present elasto-mammography simulations using numerical breast phantoms containing tumors in Section 3. Section 4 investigates the influences of various errors with the measurements, including noise with displacements, geometric mismatch, and elastic contrast ratios. Conclusions are drawn in the last section.

### 2. METHODOLOGY OF ELASTOGRAPHY RECONSTRUCTION

In this study, the mechanical properties of normal breast tissue and tumors are assumed to be linearly elastic and isotropic; that is, they are described with elastic Lamé parameters $\lambda$ and $\mu$. Typical clinical mammography applies two or $M$ ($M \geq 2$) individual compressions on the breast so that the maximum amount of tissues can be imaged and examined from different view angles. Information about the displacements is collected from projective images, and is used in the proposed elasto-mammography for identifying the Lamé parameters of the tissues, as will be described in the next section.

The three-dimensional (3D) reconstruction algorithm for Lamé parameters is optimization based and follows our previously developed general framework [13]. The displacement and force quantities with the $I$th experiment are denoted with superscript $(I)$. Denoting for the $I$th loading the *measured* displacement field in the biomedical medium of interest $(\Omega)$ as $\mathbf{U}^{(I)}(\mathbf{x})$, and the *calculated* displacement field associated with the trial distribution of Lamé parameters $(\lambda(\mathbf{x}), \mu(\mathbf{x}))$ as $\mathbf{u}^{(I)}(\mathbf{x})$, the elasto-mammography seeks Lamé parameters such that the following objective functional $\Phi(\lambda(\mathbf{x}), \mu(\mathbf{x}))$ is optimally minimized:

$$\Phi(\lambda(\mathbf{x}), \mu(\mathbf{x})) = \sum_{I=1}^{M} \int_{\Omega} (\mathbf{u}^{(I)}(\mathbf{x}) - \mathbf{U}^{(I)}(\mathbf{x})) \cdot \boldsymbol{\chi}^{(I)}(\mathbf{x}) \cdot (\mathbf{u}^{(I)}(\mathbf{x}) - \mathbf{U}^{(I)}(\mathbf{x}))\, dV, \tag{1}$$

where the second-order tensor $\boldsymbol{\chi}^{(I)}$ simply takes diagonal matrix form, that is, $(\boldsymbol{\chi}^{(I)}(\mathbf{x}))_{ij} = \delta_{ij}\omega_i^{(I)}(\mathbf{x})$ ($I = 1, 2, \ldots, M$; $i, j = 1, 2, 3$). The weight function $\omega_i^{(I)}(\mathbf{x})$ equals zero if the $i$th displacement component is *not* measured at point $\mathbf{x}$. To include the surface displacement as measurement, $\omega_i^{(I)}(\mathbf{x})$ is considered as a generalized function on the boundary of $\Omega$.

The elasto-mammography reconstruction follows an iterative optimization procedure, as schematically shown in Figure 1. We employ a large-scale limited-memory BFGS (L-BFGS) optimization method [14], which requires user-supplied gradients of the objective functional, that is, $\partial\Phi/\partial\lambda$ and $\partial\Phi/\partial\mu$. Continuum formulas for the gradients have been derived by Oberai et al. [15] for isotropic elastography and by Liu et al. [13] for general anisotropic cases. Here, we give the finite-element presentations for the objective functional $\Phi$ and its gradients.

Following standard finite-element procedures (e.g., [16]), the displacement field $\mathbf{u}^{(I)}(\mathbf{x})$ is discretized as vector $u^{(I)}$, which satisfies the equilibrium equation

$$K u^{(I)} = F^{(I)} \quad (I = 1, 2, \ldots, M), \tag{2}$$

where vector $F^{(I)}$ represents the nodal force. Once finite-element mesh is generated and discretization method is selected, the stiffness matrix $K$ depends only on the Lamé parameters $(\lambda(\mathbf{x}), \mu(\mathbf{x}))$. Consistently, the *measured* displacement field $\mathbf{U}^{(I)}(\mathbf{x})$ is discretized as vector $U^{(I)}$. Therefore, the

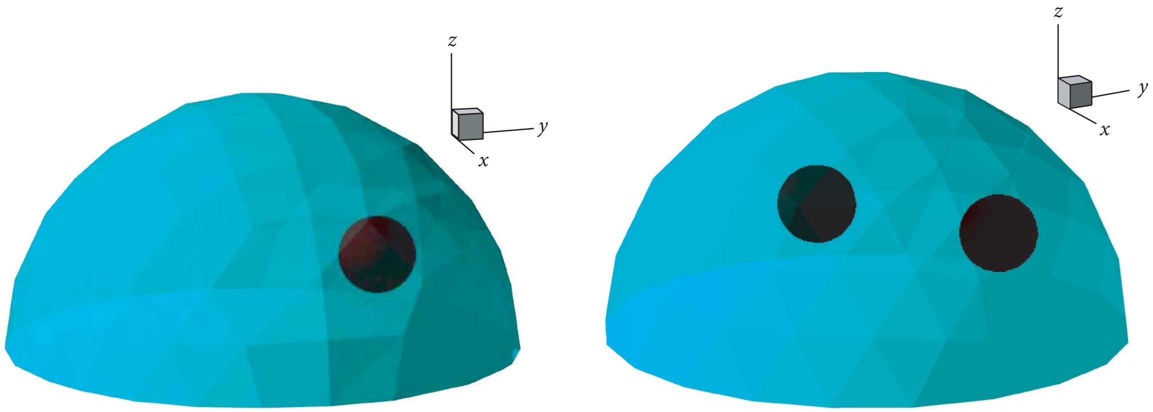

FIGURE 2: 3D phantoms mimicking the normal breast tissue and embedded tumor(s). Finite-element mesh is shown on the external surface. (a) Phantom I, with one tumor; (b) Phantom II, with two tumors.

objective functional (1) reads

$$\Phi(\lambda(\mathbf{x}),\mu(\mathbf{x})) = \sum_{I=1}^{M}\left(u^{(I)} - U^{(I)}\right)^T X^{(I)}\left(u^{(I)} - U^{(I)}\right), \quad (3)$$

in which the matrix $X^{(I)}$ corresponds to the weight function $\chi^{(I)}(\mathbf{x})$ and has the same dimension as the stiffness matrix $K$. It has been shown [13, 15] that the gradients of $\Phi$ can be calculated conveniently via

$$\delta\Phi = \sum_{I=1}^{M}\left(u^{(I)}\right)^T \delta K w^{(I)}, \quad (4)$$

where the *adjoint* displacement $w^{(I)}$ is the solution of

$$K w^{(I)} = -2X^{(I)}\left(u^{(I)} - U^{(I)}\right) \quad (I = 1, 2, \ldots, M). \quad (5)$$

It is noted that $u^{(I)}$ and $w^{(I)}$ share the same Cholesky factorization (e.g., [16]) for the stiffness matrix $K$, thus the computational expense for solving $w^{(I)}$ (5) is minimal once $u^{(I)}$ is solved (2).

In the proposed elasto-mammography technique, anatomic structures of the normal breast tissue and tumor are prescanned. Therefore the breast can be modeled as a piecewise homogenous medium, with uniform Lamé parameters $(\lambda_{\text{tissue}}, \mu_{\text{tissue}})$ for the normal breast tissue region and uniform parameters $(\lambda_{\text{tumor}}, \mu_{\text{tumor}})$ for the tumor region. Consequently, there are four gradients to be calculated:

$$\begin{aligned}\frac{\partial\Phi}{\partial\lambda} &= \sum_{I=1}^{M}\sum_{N}\left(u_e^{(I)}\right)_N^T \frac{\partial (K_e)_N}{\partial\lambda}\left(w_e^{(I)}\right)_N,\\ \frac{\partial\Phi}{\partial\mu} &= \sum_{I=1}^{M}\sum_{N}\left(u_e^{(I)}\right)_N^T \frac{\partial (K_e)_N}{\partial\mu}\left(w_e^{(I)}\right)_N,\end{aligned} \quad (6)$$

in which the inner summations are taken over all the elements in the tissue region for the gradients $\partial\Phi/\partial\lambda_{\text{tissue}}$ and $\partial\Phi/\partial\mu_{\text{tissue}}$, and over all the elements in the tumor region for the gradients $\partial\Phi/\partial\lambda_{\text{tumor}}$ and $\partial\Phi/\partial\mu_{\text{tumor}}$. In (6), $(K_e)_N$ is the element stiffness matrix of the $N$th element, and $(u_e^{(I)})_N$ and $(w_e^{(I)})_N$ are the element nodal displacements with the $I$th loading. It is also noted that $\partial(K_e)_N/\partial\lambda$ and $\partial(K_e)_N/\partial\mu$ are constant matrices for the $N$th element.

### 3. NUMERICAL SIMULATIONS

In this section, simulations are performed with numerical breast phantoms to identify the elastic parameters for normal tissue and tumor(s). The 3D breast phantoms contain one and two tumors, respectively. To simulate mammography compression, two types of loadings are applied, respectively, on the phantoms from different loading angles. Surface forces and part of the boundary displacements are extracted from the forward computation results, in compliance with the capability of projective imaging, and are used as input for the reconstruction. In the following text, the units are "cm" for length and displacements, "kPa" for elastic moduli, and "kN" for nodal forces.

### 3.1. Forward computations

Let us first consider a 3D phantom consisting of a half-spherical matrix with an embedded spherical inclusion (Figure 2(a)). The soft matrix, 10 cm in diameter and center at $(x, y, z) = (0, 0, 0)$, imitates normal breast tissue. The hard inclusion, 1.5 cm in diameter and center at $(2, 1.75, 2.25)$, simulates a tumor. The second phantom (Figure 2(b)) is similar, but has one more tumor of the same size and center at $(-1.8, 0, 2)$. We denote these phantoms as "Phantom I" and "Phantom II," respectively. The phantoms are discretized with standard 3D tetrahedral elements. Phantom I consists of 1114 nodes and 6070 elements, while Phantom II consists of 1657 nodes and 9340 elements.

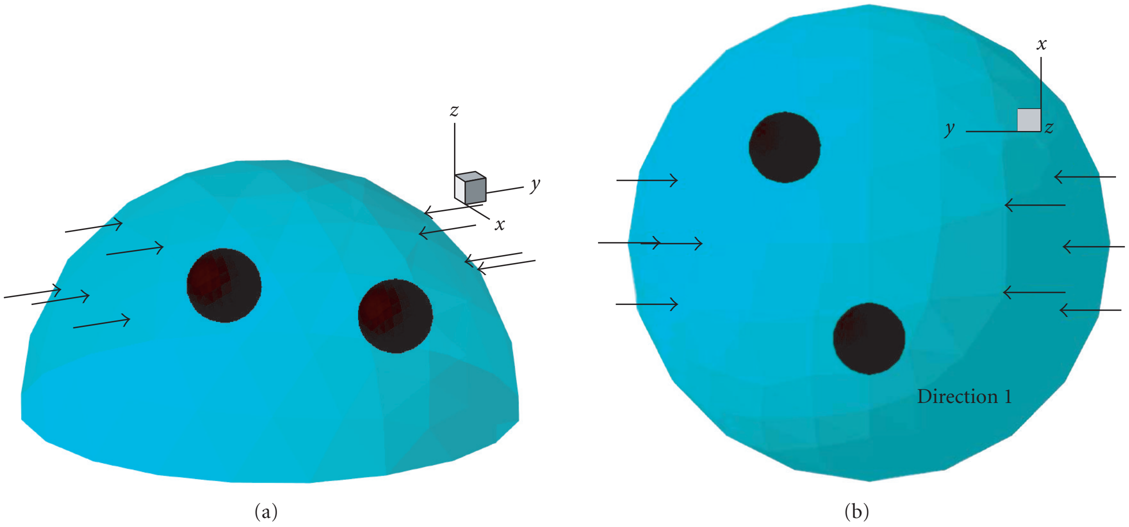

FIGURE 3: Loading 1: compression nodal force applied on the surface of Phantom II. (a) 3D view; (b) $x$-$y$ plane view to show direction 1.

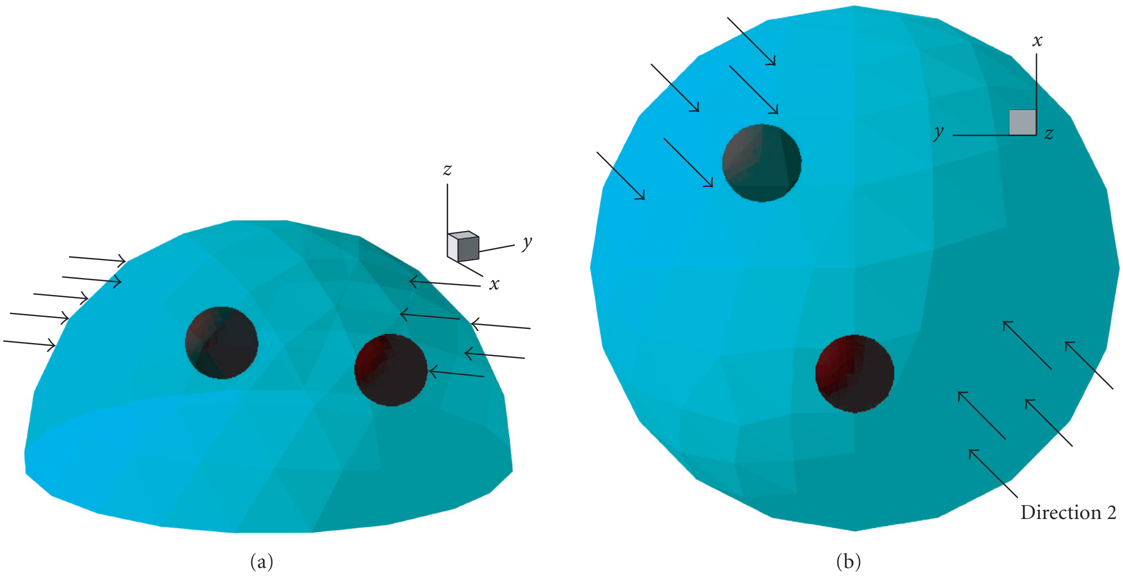

FIGURE 4: Loading 2: compression nodal force applied on the surface of Phantom II. (a) 3D view; (b) $x$-$y$ plane view to show direction 2.

The materials are assumed isotropic. The Lamé parameters $(\lambda, \mu)$ are $(25, 7.5)$ for the soft breast tissue and are $(125, 25)$ for the tumor. Young's modulus $E$ and Poisson's ratio $\nu$ are related to Lamé parameters via

$$E = \frac{\mu(3\lambda + 2\mu)}{\lambda + \mu}, \qquad \nu = \frac{\lambda}{2(\lambda + \mu)},$$
$$\lambda = \frac{E\nu}{(1+\nu)(1-2\nu)}, \qquad \mu = \frac{E}{2(1+\nu)}. \tag{7}$$

Hence, $(E, \nu)$ are $(20.769,\ 0.384\,62)$ for soft tissue and $(70.833, 0.416\,67)$ for tumor. Note that the tumor is assumed approximately 3.5 times as stiff as the surrounding tissue. In general, a tumor is much stiffer than the surrounding normal tissues. However, the ratio between the stiffness of cancerous and normal breast tissues found in the literature shows variations from a few times to a few ten times [4]. Skovoroda et al. [5] recognized that this is partially due to the nonlinearity effect in which the apparent stiffness increases with the strain applied. Effects of the contrast ratio on elastomammography will be discussed later.

In the simulations, the displacements are zero on the base surface where $z = 0$. Two compression loadings are applied on the upper surface of breast phantoms, respectively. For Loading 1, nodal force of 0.005 kN is applied on some of the surface nodes, as plotted in Figure 3 for Phantom II. Loading 2 applies nodal force $|F_x| = |F_y| = 0.004$ kN on the other set of surface nodes, as shown in Figure 4. Note that the loading

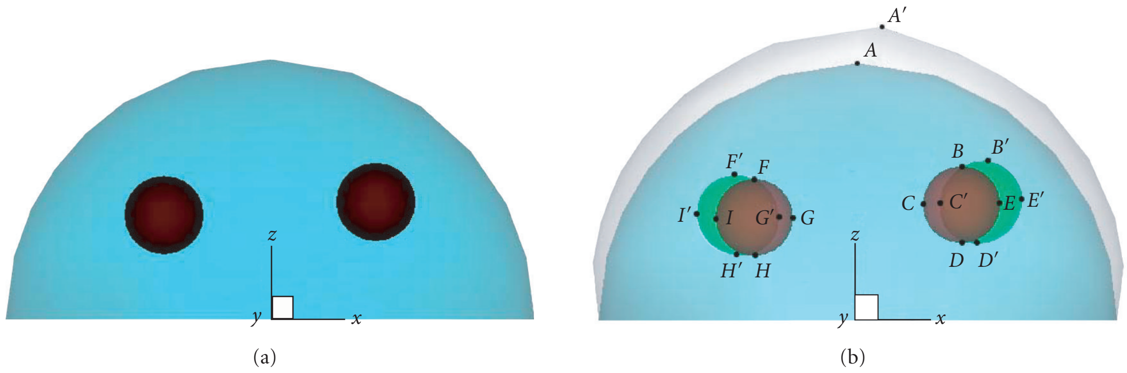

FIGURE 5: Mammography projections for Phantom II under Loading 1. Projections are made in direction 1. (a) Undeformed projection; (b) deformed projection overlaps on undeformed projection. In the projections, vertexes $A \sim I$ in undeformed projection move to $A' \sim I'$ in deformed projection, respectively.

directions are different by $\pi/4$. For convenience, we denote the direction with Loading 1 as "direction 1," and that with Loading 2 as "direction 2."

### 3.2. Data acquisition

Given the Lamé parameters for normal breast tissue and tumor(s), the deformations in response to the external loadings 1 and 2 are obtained by solving finite element (2), respectively. First of all, the external surface of a breast at undeformed state can be reconstructed from images taken with a 3D camera (e.g., [17]). Then, for each external loading, two mammography projections are made in the compression direction; that is, one projection with undeformed state and one with deformed configuration. The shape and location of the tumor(s) can further be estimated from the undeformed projections along different orientations. It is recognized that real tumors may be irregular in shape and difficult to reconstruct accurately with limited number of projections. As a first-order approximation, we assume that tumors are spherical initially, and deform into ellipsoids. The initial size and center of tumors are readily estimated with two undeformed projections made in different directions. For instance, directions 1 and 2 in the present simulations, as plotted in Figures 5(a) and 6(a) for Phantom II. Note that Phantom I is considered as Phantom II with absence of the tumor initially at $(-1.8, 0, 2)$.

We extract displacement information from projection of deformed configurations. Based on the micromechanics theory for deformation of an inclusion in a large medium (e.g., [18]), it is reasonable to estimate that an initially spherical tumor deforms into an ellipsoid. Because of the relatively simple uniaxial compression loadings applied in mammography, it is further approximated that vertexes of an object in an undeformed projection remain vertexes in the corresponding projection after compression deformation. For example, in Figure 5(b) for Loading 1, point $A$ is the top vertex of tissue in undeformed projection. It moves to vertex $A'$ after deformation. Points $B \sim I$ are vertexes of the tumors in undeformed projection in direction 1. They displace to vertexes $B' \sim I'$, respectively. Thus, by measuring the vertex locations in projections before and after deformation, their displacement information can be obtained. For example, displacement components $u_x$ and $u_z$ in Loading 1 for vertexes $A \sim I$ are extracted from the projections as in Figure 5. Acquisition of displacement information with Loading 2 makes use of projections, see Figures 6(a) and 6(b), and follows the same procedure. It is noted that the two tumors partly overlap in the projections in direction 2, and vertexes $C$ and $G$ are in shadow. For such a case, the vertex displacements are still attainable according to the grey density information in the projections with loss of some accuracy. The collected displacement data are denoted as $U^{(1)}$ and $U^{(2)}$ for elasto-mammography reconstruction.

Accurate displacement measurement with high spatial resolution will benefit elastography reconstruction in general. However, pinpoint tracking of large number of material points in an object is still a challenge in medical imaging [19], in particular for simple mammography projections that lack natural landmarks. Therefore, we propose elasto-mammography that only makes use of displacements of a few special points extracted directly from projections. As described above, the points include top vertex on the upper breast surface ($A$ in Figure 5(b)) and vertexes of the tumors in projections ($B \sim I$). Displacements measured at other points, for instance, on the external surface with a 3D camera, should enhance the efficiency and accuracy of elasto-mammography.

### 3.3. Ideal elasto-mammography

With the described data acquisition method, displacements at some key points are extracted from deformed and undeformed projections with the two compression loadings, and are used as measurements $U^{(1)}$ and $U^{(2)}$ for elasto-mammography reconstruction. Compression nodal forces applied on the surface are also known with the loadings. Given initial estimate, the Lamé parameters for tissue and tumor are reconstructed following our optimization procedure (Figure 1).

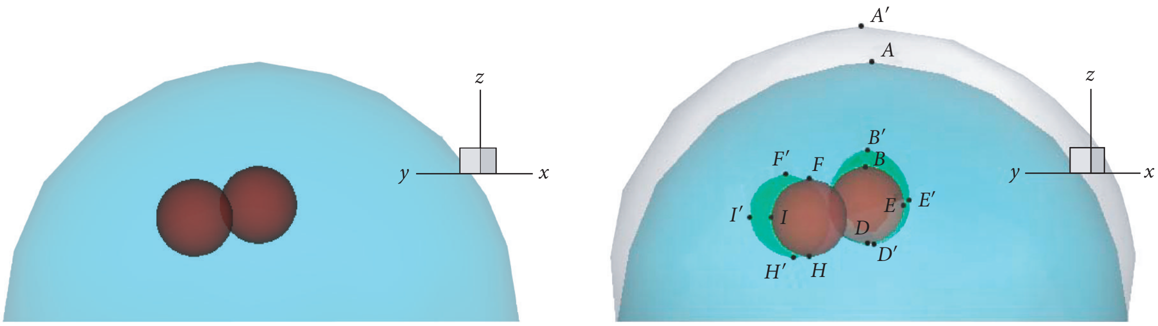

Figure 6: Mammography projections for Phantom II under Loading 2. Projections are made along direction 2. (a) Undeformed projection; (b) deformed projection overlaps on undeformed projection. In the projections, vertexes $A \sim I$ in undeformed projection move to $A' \sim I'$ in deformed projection, respectively.

Table 1: Initial estimate and reconstructed results for elasto-mammography.

| | Tissue | | | | Tumor | | | |
|---|---|---|---|---|---|---|---|---|
| | $\lambda$ | $\mu$ | $E$ | $\nu$ | $\lambda$ | $\mu$ | $E$ | $\nu$ |
| Real | 25 | 7.5 | 20.769 | 0.384 62 | 125 | 25 | 70.833 | 0.416 67 |
| Estimate | 11 | 194.5 | 399.441 | 0.026 76 | 333 | 33.5 | 97.705 | 0.454 30 |
| | Reconstruction results | | | | | | | |
| Ideal I | 24.999 | 7.5 | 20.769 | 0.384 62 | 124.817 | 24.999 | 70.815 | 0.416 58 |
| Ideal II | 25.012 | 7.5 | 20.764 | 0.384 69 | 125.219 | 25.038 | 70.947 | 0.416 79 |
| Noise I | 25.155 | 7.495 | 20.764 | 0.385 22 | 106.750 | 25.055 | 70.404 | 0.404 95 |
| Noise II | 26.048 | 7.503 | 20.82 | 0.388 19 | 146.801 | 22.379 | 64.176 | 0.433 86 |
| Mismatch I | 24.972 | 7.502 | 20.777 | 0.384 49 | 129.398 | 24.901 | 70.929 | 0.419 06 |
| Mismatch II | 25.042 | 7.493 | 20.703 | 0.385 08 | 155.273 | 26.943 | 78.826 | 0.462 83 |

The ideal case is considered first; that is, the displacements, geometry, and compression nodal forces are exactly measured, and are used as input for reconstruction. Rows "Ideal I" and "Ideal II" of Table 1 give the reconstruction results for Phantom I and Phantom II, respectively. Convergent loci of the Lamé parameters $(\lambda, \mu)$ are plotted in Figure 7. The loci for Phantom I (Figure 7(a)) and Phantom II (Figure 7(b)) are very similar. It is observed that $(\lambda, \mu)$ of the tissue approach the real value rapidly. After about 20 iteration steps, their relative errors are well within the range of 5%. Then they experience some minor adjustment. In contrast, Lamé parameters of the tumor converge slower, in particular for $\lambda$, which starts to fall to the real value after about 40 steps. After about 50 steps, all parameters are accurately identified, with the largest error of about $\pm 0.18\%$ (for $\lambda$ of tumor). Reconstructions using different initial estimates have been conducted. Very similar convergent profiles are found for the parameters, and highly accurate results are obtained. This indicates efficiency and uniqueness of the proposed elasto-mammography using projective measurements.

The slower convergent speed of Lamé parameters of the tumor, in particular for $\lambda$, is explained by the roles they play in the deformation due to the applied loadings, as discussed by Liu et al. [13]. In general, parameters with the most significant influence on the deformation are also those that are most accurately and easily identified. The influence of a parameter depends on size and location of the material region it belongs to, as well as characteristics of the deformation. For the present simulations, $\lambda$ and $\mu$ of the tissue are dominant, while those of tumor are much less influential, due to the small size and deep location of the tumor(s). Slower convergence of $\lambda$ for tumor indicates that the present loadings do not introduce enough volumetric strain in the tumor.

### 4. DISCUSSION

### 4.1. Effect of noise

The above elasto-mammography reconstructions are conducted using ideal inputs. In practice, several factors will affect the performance of elasto-mammography, the most common one among which is the noise with displacement measurement. To investigate the capability of the proposed elasto-mammography modality and algorithm to handle imperfect real data due to inevitable measurement errors, we conduct reconstruction using noisy input; that is, each component of $U^{(1)}$ and $U^{(2)}$ is added with a randomly selected relative error between $-5\%$ and 5%.

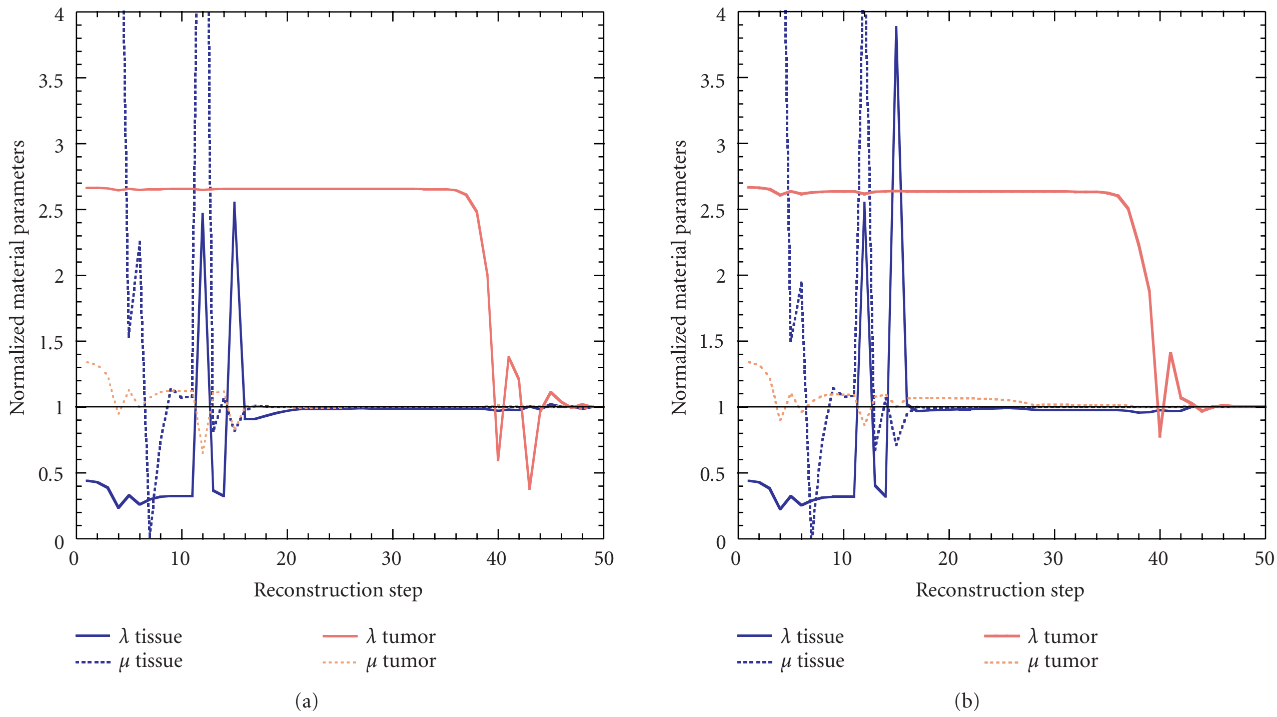

FIGURE 7: Convergent loci of elasto-mammography reconstruction for Lamé parameters $(\lambda, \mu)$ of normal breast tissue and tumor, normalized with the exact values correspondingly. No measurement error is considered. (a) Phantom I (one tumor); (b) Phantom II (two tumors).

The results are shown as "Noise I" (for Phantom I) and "Noise II" (for Phantom II) in Table 1, and the convergent loci are plotted in Figure 8. The overall convergent loci are very similar to the "ideal" cases. Lamé parameters $(\lambda, \mu)$ of the tissue need about 20 steps to approach closely to the real values, while those of the tumor need about 50 steps for convergence. The tissue parameters are very accurately identified, with the largest relative error of 4% for $\lambda$ of Phantom II, and errors well within $\pm 1\%$ for the others. The Lamé parameters $(\lambda, \mu)$ of tumor, however, are not as robust, with relative errors of $(-14.6\%, 0.22\%)$ and $(17.4\%, -15.5\%)$ for Phantom I and Phantom II, respectively. In spite of these reconstruction errors, it is still positive that the elasto-mammography results are accurate enough for diagnosis of tumors, noting the significant differences of stiffness between normal tissue, and benign and malignant tumors (e.g., [3–5]). The better robustness of the tissue parameters is also explained by the strong roles they play in the deformation, as we have discussed above. Furthermore, as suggested by Liu et al. [13], multiple sets of well-designed loadings should help to bring out the influences of all the material parameters, and thus suppress the effects of noise.

### 4.2. *Effect of geometry mismatch*

Another concern for elasto-mammography is the geometric depiction of the tumor. As described in the section of data acquisition, we use a simple sphere to approximate a real tumor, and estimate its size and location from two undeformed mammography projections. This inevitably introduces geometric mismatch for practical elasto-mammography. To investigate the effect of geometry mismatch, the two phantoms are redesigned by replacing the spherical tumors with cubic tumors. Note that the edge length of the cube is $3/\sqrt{5}$ cm. Forward simulations are conducted under the same Loading 1 and Loading 2 with the new phantoms. Then, mammography projections are made of the new undeformed and deformed configurations. To extract geometric and displacement data from the projections, we still use spherical approach. As schematically shown in Figure 9, a cubic tumor is approximated with a spherical one, whose size and location are determined by the two undeformed projections in direction 1 and direction 2. Then, the estimated spherical tumors are used for elasto-mammography reconstruction of the material parameters. The results are shown as "Noise I" (for Phantom I) and "Noise II" (for Phantom II) in Table 1. Convergent loci are found to be similar to the previous cases, and are not shown.

The tissue parameters again show excellent robustness. The geometric mismatch introduces relative errors less than 0.17%. Due to the relatively small size of the tumor(s), their Lamé parameters $(\lambda, \mu)$ are more sensitive to geometric mismatch, with relative errors $(3.52\%, 0.40\%)$ for Phantom I and $(24.2\%, 7.78\%)$ for Phantom II. In comparison to the displacement noise, the geometry mismatch seems to have slightly less overall influence on the reconstruction results. However, this point is based on the current phantoms, and needs further investigation.

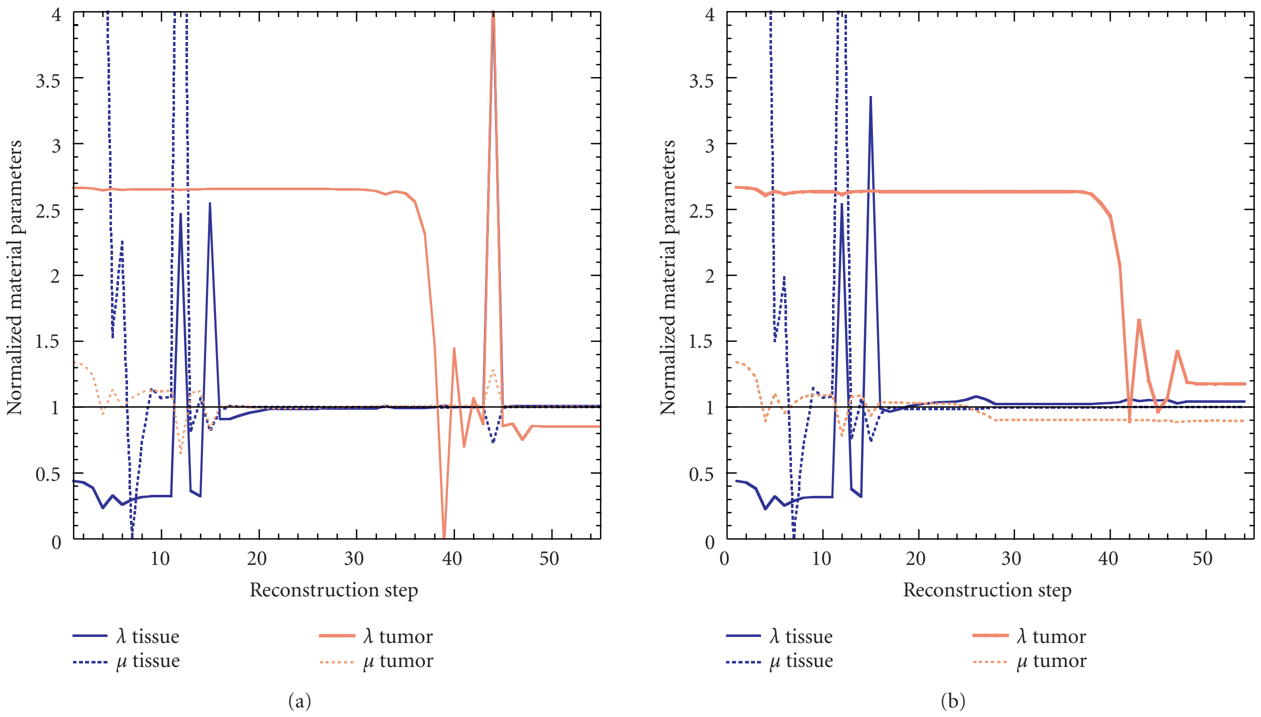

FIGURE 8: Convergent loci of elasto-mammography reconstruction for Lamé parameters $(\lambda, \mu)$ of normal breast tissue and tumor, normalized with the real values correspondingly. Noise is considered. (a) Phantom I (one tumor); (b) Phantom II (two tumors).

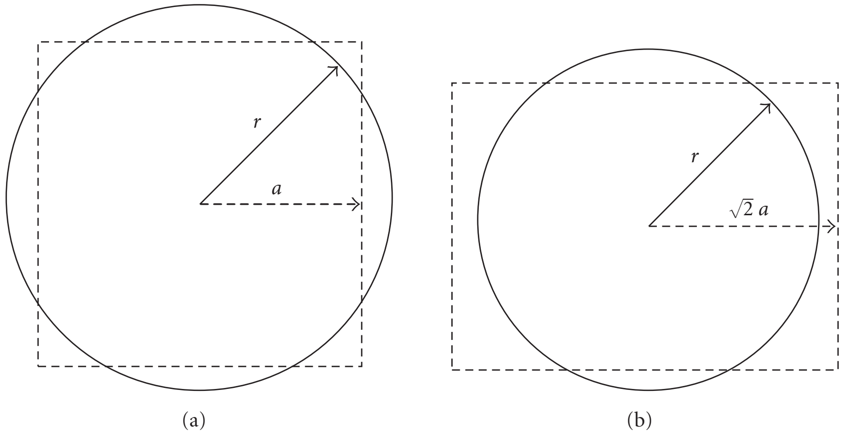

FIGURE 9: Geometry mismatch. A sphere is used to approximate a real cubic tumor. Size and location of the sphere are determined from projections of the cubic tumor in direction 1 (a) and direction 2 (b). $r/a = \sqrt{5}/2$.

In this study, a perfect sphere is assumed to simulate the real tumor. From this simplified model, the information of deformation can be easily obtained from the projections. However, investigation of geometry mismatch is needed since most of real tumors have irregular shapes. A cube (a rectangle or square in projections) is used to represent a real tumor, and a sphere (a circle in projections) approximates it. From Figure 9(a), it can be seen that most of the areas between circle and square overlap. The results demonstrate that geometry mismatch does not have a great influence on this reconstruction. It is therefore suggested that, for an irregular shape of a real tumor in projections, we could choose a circle to approximate it and do reconstruction based on this simplified model.

### 4.3. *Effect of contrast ratio*

The material stiffness is a key feature that distinguishes benign from malignant tumors [4–6]. Contrast ratio, defined as the ratio between Young's modulus of tumor and normal

Table 2: Elasto-mammography simulation using phantoms with different stiffness contrast ratios.

| | Tissue | | | | Tumor | | | |
|---|---|---|---|---|---|---|---|---|
| | $\lambda$ | $\mu$ | $E$ | $\nu$ | $\lambda$ | $\mu$ | $E$ | $\nu$ |
| | Contrast ratio = 1.5 | | | | | | | |
| Real | 25 | 7.5 | 20.769 | 0.384 62 | 54.979 | 10.995 | 31.154 | 0.416 67 |
| Phantom I | 25 | 7.5 | 20.769 | 0.384 61 | 54.979 | 10.995 | 31.154 | 0.416 67 |
| Phantom II | 24.928 | 7.5 | 20.766 | 0.384 36 | 55.709 | 11.025 | 31.154 | 0.417 20 |
| | Contrast ratio = 8.0 | | | | | | | |
| Real | 25 | 7.5 | 20.769 | 0.384 62 | 293.22 | 58.642 | 166.15 | 0.416 67 |
| Phantom I | 24.989 | 7.5 | 20.767 | 0.384 58 | 332.56 | 58.676 | 167.23 | 0.425 01 |
| Phantom II | 24.994 | 7.497 | 20.761 | 0.384 63 | 331.42 | 59.124 | 168.42 | 0.424 31 |

breast tissue, covers a wide range. For benign tumors, contrast ratio typically varies from 2.0 to about 5.0. For malignant tumors, it is considerably higher. Our numerical experiments [13] indicate that accuracy of elastography reconstruction depends not only on type of loading and measurement accuracy, but also on the contrast ratio. In case that the tumor is very hard, the material parameters may be identified qualitatively, but not quantitatively.

To investigate the effect of contrast ratio, we conducted elasto-mammography reconstructions with soft and hard phantoms, whose Lamé parameters $(\lambda, \mu)$ are set to create contrast ratios (CR) of 1.5 and 8.0, respectively. Table 2 gives the real Lamé parameters and reconstruction results. Compared to the previous case with CR about 3.5 (Table 1, "Ideal I" and "Ideal II"), results for the soft phantoms (CR = 1.5) are even more accurate, in particular for $\lambda$ of the tumor. For the hard phantoms (CR = 8.0), the tissue parameters are also exact; however, $\lambda$ of tumor carries relative reconstruction errors of about 13% for both phantoms, which is considerably larger than the soft cases with CR = 3.5 and 1.5. The reason is that deformation of a relatively softer tumor is larger than a hard one, and thus is more sensitive to small variation of its material parameters. Also as discussed above, $\lambda$ of the tumor seems to have the least influence on the specific deformations considered in the simulations. On the other hand, it is convincing that the proposed elasto-mammography is efficient in revealing the contrast ratio and telling whether a tumor is malignant or benign. In general, a tumor is suspected of malignancy when the contrast ratio is higher than 6. In the present simulations, when the "real" contrast ratio is 8.0, the elasto-mammography reconstruction yields 8.11, which is fully acceptable for the diagnostic purpose.

The elastography simulations of Liu et al. [13] assumed tumor and normal breast tissue as general anisotropic materials, and applied four sets of loading on a breast phantom to bring out all the elastic parameters. Their isotropic simulation suggested that displacements measured from a single loading are adequate for unique identification of the Lamé parameters $(\lambda, \mu)$ of tissue and tumor. However, their measurement includes displacement on the entire external surface and the tissue-tumor interface of a breast, requiring more complex imaging equipment. In our elasto-mammography proposal, displacement measurement has been reduced to a few vertexes, and can be readily obtained from simple mammography projections. A tradeoff is that two or more sets of compression loadings may be needed to obtain adequate identification.

Mathematical proof for uniqueness results of elasto-mammography using projection measurements is yet under further investigation. Our simulations always yield the same material parameters (within the numerical processing errors), regardless of the initial estimate. With ideal measurements, the resulting parameters exactly match the real values specified for the models. When displacement noise and geometry mismatch are taken into consideration, the resulting parameters have reconstruction errors, however, are close enough to their real values for application purpose. In summary, the proposed elasto-mammography method is numerically stable and robust, is relatively simple to perform, and thus has great potential for clinical applications.

### 5. CONCLUSIONS

A new method that combines elastography and mammography to reconstruct the elastic field of the breast is reported. Displacement and geometry measured from deformed and undeformed mammography projections are applied as input data to reconstruct the isotropic material parameters for normal breast tissue and tumor. Our numerical simulations demonstrate that unique and accurate results can be obtained using information extracted from only two sets of projections. Displacement noise, geometry mismatch, and material contrast ratio do not adversely affect the results, demonstrating that our method is stable and robust. These findings are sufficiently encouraging to warrant both further development and clinical evaluation of our reconstruction method.

### ACKNOWLEDGMENTS

This work is partially supported by the University of Iowa Informatics Initiative Grant and the US Army's Breast Cancer Research Program Concept Award (W81XWH-05-1-0461).

### REFERENCES

[1] S. Muller, "Full-field digital mammography designed as a complete system," *European Journal of Radiology*, vol. 31, no. 1, pp. 25–34, 1999.

[2] P. J. Kornguth and R. C. Bentley, "Mammographic-pathologic correlation: Part 1. Benign breast lesions," *Journal of Women's Imaging*, vol. 3, no. 1, pp. 29–37, 2001.

[3] A. P. Sarvazyan, A. R. Skovoroda, S. Y. Emelianov, et al., "Biophysical bases of elasticity imaging," in *Acoustical Imaging*, vol. 21, pp. 223–240, Plenum Press, New York, NY, USA, 1995.

[4] P. Wellman, R. Howe, E. Dalton, and K. A. Kern, "Breast tissue stiffness in compression is correlated to histological diagnosis," Tech. Rep., Harvard BioRobotics Laboratory, Harvard University, Cambridge, Mass, USA, 1999.

[5] A. R. Skovoroda, A. N. Klishko, D. A. Gusakian, et al., "Quantitative analysis of mechanical characteristics of pathologically altered soft biological tissues," *Biofizika*, vol. 40, no. 6, pp. 1335–1340, 1995.

[6] J. Ophir, I. Céspedes, H. Ponnekanti, Y. Yazdi, and X. Li, "Elastography: a quantitative method for imaging the elasticity of biological tissues," *Ultrasonic Imaging*, vol. 13, no. 2, pp. 111–134, 1991.

[7] J. Ophir, S. K. Alam, B. Garra, et al., "Elastography: ultrasonic estimation and imaging of the elastic properties of tissues," *Proceedings of the Institution of Mechanical Engineers, Part H: Journal of Engineering in Medicine*, vol. 213, no. 3, pp. 203–233, 1999.

[8] R. Souchon, L. Soualmi, M. Bertrand, J.-Y. Chapelon, F. Kallel, and J. Ophir, "Ultrasonic elastography using sector scan imaging and a radial compression," *Ultrasonics*, vol. 40, no. 1–8, pp. 867–871, 2002.

[9] R. Muthupillai, D. J. Lomas, P. J. Rossman, J. F. Greenleaf, A. Manduca, and R. L. Ehman, "Magnetic resonance elastography by direct visualization of propagating acoustic strain waves," *Science*, vol. 269, no. 5232, pp. 1854–1857, 1995.

[10] A. Manduca, T. E. Oliphant, M. A. Dresner, et al., "Magnetic resonance elastography: non-invasive mapping of tissue elasticity," *Medical Image Analysis*, vol. 5, no. 4, pp. 237–254, 2001.

[11] D. B. Plewes, J. Bishop, A. Samani, and J. Sciarretta, "Visualization and quantification of breast cancer biomechanical properties with magnetic resonance elastography," *Physics in Medicine and Biology*, vol. 45, no. 6, pp. 1591–1610, 2000.

[12] A. Samani, J. Bishop, and D. B. Plewes, "A constrained modulus reconstruction technique for breast cancer assessment," *IEEE Transactions on Medical Imaging*, vol. 20, no. 9, pp. 877–885, 2001.

[13] Y. Liu, L. Z. Sun, and G. Wang, "Tomography-based 3-D anisotropic elastography using boundary measurements," *IEEE Transactions on Medical Imaging*, vol. 24, no. 10, pp. 1323–1333, 2005.

[14] D. C. Liu and J. Nocedal, "On the limited memory BFGS method for large scale optimization," *Mathematical Programming*, vol. 45, no. 1–3, pp. 503–528, 1989.

[15] A. A. Oberai, N. H. Gokhale, and G. R. Feijóo, "Solution of inverse problems in elasticity imaging using the adjoint method," *Inverse Problems*, vol. 19, no. 2, pp. 297–313, 2003.

[16] T. Belytschko, W. K. Liu, and B. Moran, *Nonlinear Finite Elements for Continua and Structures*, John Wiley & Sons, New York, NY, USA, 2000.

[17] D. Page, A. Koschan, S. Voisin, N. Ali, and M. Abidi, "3D CAD model generation of mechanical parts using coded-pattern projection and laser triangulation systems," *Assembly Automation*, vol. 25, no. 3, pp. 230–238, 2005.

[18] J. D. Eshelby, "The determination of the elastic field of an ellipsoidal inclusion, and related problems," *Proceedings of the Royal Society of London. Series A*, vol. 241, no. 1226, pp. 376–396, 1957.

[19] J. V. Hajnal, D. L. G. Hill, and D. J. Hawkes, Eds., *Medical Image Registration*, CRC Press, Boca Raton, Fla, USA, 2001.

**Z. G. Wang** is currently a Ph.D. student in the Department of Civil and Environmental Engineering at the University of Iowa, conducting research on biomedical elastography for breast cancer diagnosis under supervision of Drs. L. Z. Sun and G. Wang. He obtained his B.S. degree from Peking University in 1999, and obtained his M.S. degree from the Chinese Academy of Sciences in 2002.

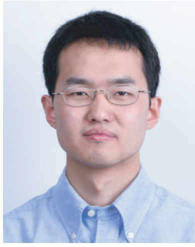

**Y. Liu** is a Research Scientist in the Center for X-Ray and Optical Tomography at the University of Iowa. He received his Ph.D. degree in mechanical engineering from the University of Pennsylvania in 2003. His research interests include biomedical imaging and elastography for breast cancer and myocardial abnormality.

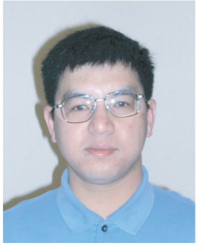

**L. Z. Sun** is an Associate Professor in the Department of Civil and Environmental Engineering at the University of California, Irvine (UCI). He has a secondary appointment of Associate Professor in UCI Chemical Engineering and Materials Science. Before joining UCI in 2005, he was an Assistant and later an Associate Professor at the University of Iowa. He received his Ph.D. degree in structural mechanics from the University of California, Los Angeles (UCLA), in 1998. His main area of research interest is the micro/nanomechanics of heterogeneous composite materials, with applications for civil, mechanical, aerospace, electronic, and biomedical engineering. He has published more than 100 papers including 50 peer-reviewed journal papers in the fields of mechanics and materials, applied physics, and biomedical engineering. He has organized 10 symposia for various societies. He served as a reviewer for more than 25 journals and 5 funding agencies. He is a Guest Editor for the International Journal of Damage Mechanics and an Associate Editor for the International Journal of Biomedical Imaging. He is a Member of ASCE, ASME, MRS, and AAAS.

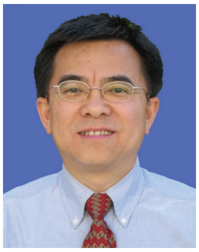

**G. Wang** received the B.E. degree in electrical engineering from Xidian University, Xian, China, in 1982, M.S. degree in remote sensing from Graduate School of Academia Sinica, Beijing, China, in 1985, and M.S. and Ph.D. degrees in electrical and computer engineering from State University of New York, Buffalo, in 1991 and 1992. He was Instructor and Assistant Professor in the Department of Electrical Engineering, Graduate School of Academia Sinica, from 1984–1988,

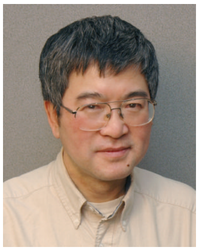

Instructor and Assistant Professor in the Mallinckrodt Institute of Radiology, Washington University, St. Louis, Mo, from 1992–1996. He was an Associate Professor at the University of Iowa from 1997–2002. Currently, he is a Professor in the Departments of Radiology, Biomedical Engineering, Mathematics, Civil Engineering, and Electrical and Computer Engineering, and the Director of the Center for X-Ray and Optical Tomography, University of Iowa. His interests include computed tomography, bioluminescence tomography, and systems biomedicine. He has published over 300 journal articles and conference papers, including the first paper on spiral/helical cone-beam CT and the first paper on bioluminescence tomography. He is the Editor-in-Chief of the International Journal of Biomedical Imaging, and the Associate Editor for the IEEE Trans. Medical Imaging and Medical Physics. He is an IEEE Fellow and an AIMBE Fellow. He has also received a number of awards for academic achievements.

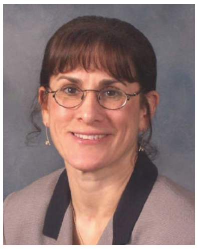

**L. L. Fajardo** is a Professor and Chair of Radiology Department at the University of Iowa since 2002. She earned her Medical degree from Pritzker School of Medicine, University of Chicago, in 1984. She completed her residency in diagnostic radiology at the University of Arizona and joined the faculty there in 1990. She was the Head of the Division of Mammography and Breast Imaging at the University of Arizona between 1990 and 1994. She then joined the Department of Radiology, University of Virginia, as the Vice Chair of Research and served in that position for five years before moving to Johns Hopkins Medical Institutions in 1999. Her clinical practice and research efforts focus on mammography and digital imaging, and she has published extensively in these areas. She also serves in an editorial capacity for several academic journals and is active with various professional organizations.

